# Can I Have a Bite? The Influence of Infant Begging on Food Sharing in Capuchin Monkeys (*Sapajus Libidinosus*)

**DOI:** 10.1002/ajp.70174

**Published:** 2026-06-05

**Authors:** Julia Omena, Mábia B. Cera, Guilbert Araujo, Helena F. Lima, Patrícia Izar

**Affiliations:** ^1^ Institute of Psychology University of São Paulo São Paulo Brazil; ^2^ nstitute of Environmental, Chemistry and Pharmaceutical Sciencies Federal University of São Paulo São Paulo Brazil

**Keywords:** informational hypothesis, nutritional hypothesis, parental investment

## Abstract

In primates, food sharing from mothers to infants may serve two main functions: supporting offspring nutritional needs (nutritional hypothesis) and facilitating the acquisition of dietary knowledge, such as what and how to eat (informational hypothesis). We investigated these hypotheses in a wild population of bearded capuchin monkeys *Sapajus libidinosus*, at Fazenda Boa Vista, Brazil. We analyzed 194 h of footage of 10 infants filmed over their first 18 months of life using focal animal sampling. Mothers shared food exclusively in response to infant solicitation, with solicitation events observed across 48 mother feeding episodes. Infants were successful in obtaining food in 29 bouts but failed in 19. In 15 of the 29 successful bouts, mothers only shared food after repeated solicitations, suggesting that food sharing involves a negotiated process. We found partial support for the nutritional hypothesis: food sharing increased as the infants aged. Infants also solicited more high‐quality and hard‐to‐process food items, but food sharing did not differ according to these food categories. Additionally, mothers were less likely to share lizards. Our findings suggest that food sharing in capuchins is driven primarily by infant begging, rather than active maternal investment, highlighting the dynamic interplay between mother and infant in promoting infant development.

## Graphical abstract

Food sharing between mother and offspring in capuchin monkeys is a response to the infant's begging. Infants solicit more frequently high‐quality and hard‐to‐process foods and they are more likely to succeed when they are older and if the food item is not a lizard.

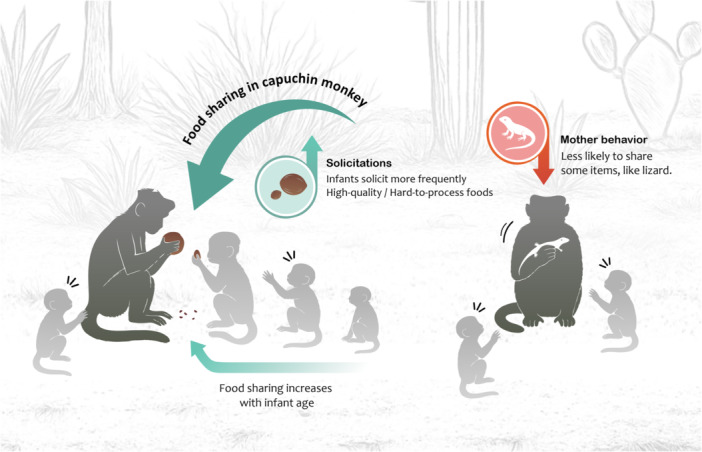

## 1 Introduction

Food provisioning is a common form of parental care in many taxa such as mammals, birds and insects (Clutton‐Brock 1991). In primates, the provisioning of food can be accomplished by the female feeding the offspring through the maternal milk or by food transferring, where the parent or caretaker allows the offspring to eat the food that they are without resisting (Brown et al. 2004). This form of food provisioning can be favored by kin selection when the costs of acquiring food are relatively low for parents, and the benefits of receiving food are high for offspring (Jaeggi and Gurven 2013).

Brown et al. (2004) suggest two hypotheses for why this behavior may enhance offspring's growth or survival: (1) the *nutritional hypothesis* states that parents' food sharing promotes weaning and accelerates offspring's growth; and (2) the *informational hypothesis* states that parents' food sharing provides information on food properties or processing techniques. In empirical studies, support for either hypothesis depends on species and context, leaving the debate unresolved. Yi et al. (2020) showed that gibbon infants solicited more novel and lower‐quality foods from their mothers, with solicitations decreasing as they aged, favoring the informational hypothesis over the nutritional one. Similarly, in golden lion tamarins, food sharing influenced juvenile foraging choices, aligning with informational hypotheses, though more palatable foods were more likely to be shared, suggesting a role for the nutritional hypothesis (Troisi et al. 2021).

The behavior of food sharing can be proactive, where the possessor gives the food to the receiver either after request or without request. This is observed in callitrichids (de A. Moura et al. 2010), apes (Liebal and Rossano 2017), and especially humans (Jaeggi and Gurven 2013). The transfers can also be passive, where the possessor allows the receiver to take the food from their reach, without resisting. This behavior is described in half of the studied primate species, including humans, apes, and Platyrrhine monkeys (de A. Moura et al. 2010; Jaeggi and Gurven 2013; Souza‐Alves et al. 2019; Yi et al. 2020). The most common form of food transfer occurs between mothers and infants (Feistner and McGrew 1989), but it is also observed between helpers and immatures, and fathers and immatures (de A. Moura et al. 2010). In humans, food sharing between kin (mothers, fathers, grandparents, and older siblings) and children is considered inseparably linked to human life history (Gurven 2004; Kaplan and Gurven 2005); and the hunting and sharing of meat, especially, is considered to have played an important role in human evolution (Isaac 1978; Gorbunova 2024).

In many cases, the processing of food items can be difficult, especially for immature individuals. Live prey, for example, requires the skill of pursuing, catching and killing another animal (Silk 1978). Immature capuchins have been observed to prey on vertebrates in the wild, although they hunt considerably less than adult males (Falótico et al. 2018; Falótico 2022). Another example of primates processing difficult food items is the use of tools in foraging. Several populations of robust capuchin monkeys in Brazil are reported to use tools (Fragaszy et al. 2004a; Ottoni and Izar 2008; Canale et al. 2009; Mannu and Ottoni 2009; Torralvo et al. 2017), most often stones to crack tough palm nuts, usually in habitats where the monkeys frequently forage on the ground (Fragaszy et al. 2004a). Notably, by eating nuts obtained through tool‐using, bearded capuchin monkeys achieve more consistent dietary intakes and increase their energy gain (Izar et al. 2022). Investigating food sharing between individuals in populations that use tools in feeding might be enlightening, because immatures learn the tooling behavior by watching proficient individuals nut cracking and often try to steal the processed nuts or forage the pieces left by the tool users (Eshchar et al. 2016), in a behavior called “scrounging” (Ottoni et al. 2005).

It is well documented that capuchin monkey infants and juveniles do scrounge, and both adults and immatures participate in feeding in the same patch (or co‐foraging; Verderane et al. 2013). However, food sharing is rarely observed, and tolerance of immatures by adults decreases as the individual ages. Capuchin monkeys *Sapajus libidinosus* have high intra‐group competition on food sources, which can usually be monopolized by dominant individuals (Verderane et al. 2013). Immatures scrounge on tool‐using sites (Fragaszy et al. 2017), and while they have motor skills like acquisition of dexterity, postural control, and combinatorial actions (Araujo et al. 2024) developed by 6 months of age, they only become proficient at tooling after several years of learning (Fragaszy et al. 2017). Increasing body mass of growing individuals (Fragaszy et al. 2016) contributes to proficiency (Resende et al. 2021), given that the hammer stones are usually almost as heavy as the adult monkeys (Liu et al. 2016). Food transfer can be another way for unskilled immatures to access highly nutritious foods processed through tool use.

In this study, we will use *S. libidinosus* as a model to test these two alternatives, the nutritional hypothesis and the informational hypothesis (Brown et al. 2004), based on observations in the wild. Capuchin monkeys start eating solid foods at 2 months of age, and weaning is completed by 18 months in this population (Omena 2022). During this period, the infants have not yet learned how to use tools for nut cracking (Fragaszy et al. 2017) and rely on proficient individuals to obtain this resource (Eshchar et al. 2016). We observed infants between two and 18 months of age to describe the events of food sharing. A reduction in the frequency of food transfer events may support the informational hypothesis (1), which proposes that, as the infant matures, the mother no longer needs to convey information regarding appropriate food choices. On the other hand, an increase in such events may support the nutritional hypothesis (2), which posits that food sharing is intensified during the weaning period, with mothers preferentially providing more nutritionally rich items. These hypotheses are not mutually exclusive, and the present study seeks to provide novel insights into the maternal investment strategies of a Platyrrhine primate species. Given the recognized importance of food sharing in human evolution (Isaac 1978; Gorbunova 2024) and in cooperative breeding strategies across many species (Burkart et al. 2018), our study provides a valuable model for comparative research with other primates and animals.

## 2 Methods

### 2.1 Study Population

In this study, we observed bearded capuchin monkeys *Sapajus libidinosus* (Parvorder Platyrrhini, Class Mammalia) (ALFARO et al. 2012), medium‐sized primates that inhabit regions of the central‐northeastern portion of Brazil. The data were collected at Fazenda Boa Vista (FBV), located in Gilbués, northeastern Brazil (9° 49′ 55″ S, 45° 20′ 38″ W). FBV is situated in an ecotone between the Cerrado and Caatinga biomes, with seasons marked by a dry period (from May to September) and a wet period (from October to April) (Spagnoletti et al. 2012).

Bearded capuchin monkeys usually form multi‐male, multi‐female groups. Females are philopatric and form a linear dominance hierarchy, with high intra‐ and inter‐group aggression closer to food sites (Verderane et al. 2013). When they are born, infants are completely dependent on their mother for survival. By the second month, they start to ingest solid foods and to go off their mother's back, and may rely on other group members for transportation (Verderane 2005). During the first 6 months of life, the infants acquire sufficient dexterity and postural control to begin walking and foraging independently (Araujo et al. 2024), but still solicit care from the mother through nursing and transportation. For *Sapajus libidinosus*, weaning starts at 6 months old, and by the 18th month, they reach the juvenile stage, being completely independent of their mother (Omena 2022; Cera et al. 2026).

Their diet is omnivorous, and it is composed of fruits, nuts, branches, roots, leaves, flowers, invertebrates, and small vertebrates. The fruit supply is varied and available throughout the year (Verderane et al. 2013). This population routinely uses stone tools to crack palm nuts (Fragaszy et al. 2004a), which significantly improves their diet quality (Izar et al. 2022).

### 2.2 Data Collection

We used the focal‐animal sampling method (Altmann 1974) to film the individuals, with observations conducted from dawn to dusk on a weekly basis. Filming occurred whenever the focal animal was visible, using a handheld digital camera operated by trained field researchers (Marcos Fonseca de Oliveira, Claudio Fonseca and Arizomar da Silva Oliveira). The monkeys were monitored from birth to the 18th month of life. All footage is stored in the database of the Laboratory of Ethology, Development, and Social Interaction (LEDIS) at the Institute of Psychology of the University of São Paulo. The data used in this study is part of a larger dataset owned and managed by P.I. For this study, we selected a subset of videos from this broader library, which is also used in other research projects and is available upon request at ledsapajus.ip@usp.br.

The groups that inhabit FBV have been habituated to the presence of the researchers since 2006, and the monkeys are individually recognized (Izar et al. 2012). We observed 10 mother‐infant dyads from group CH (Table 1). We analyzed time samples divided in 4‐week periods (or months): 2nd, 3rd, 4th, 6th, 8th, 9th, 10th, 12th, 14th, 16th, 18th. This time period covers the infancy for *Sapajus* spp. (Verderane and Izar 2019). By analyzing every other month, we were able to cover a longer period of the infants’ lives while maximizing video coding effort. We prioritized observing the first 3 months following the onset of solid food ingestion (2nd, 3rd, and 4th). Additionally, we included the ninth month in our sampling due to evidence from Verderane et al. (2019), which showed that capuchin infants in a semi‐free‐ranging group displayed behavioral regression to developmental patterns prior to the eighth month when their mothers refused to provide care.

**Table 1:** Identity, affiliation, sex, and date of birth of the 10 *sapajus libidinosus* individuals observed in the population of fazenda boa vista.

Subject	Affiliation	Infant sex	Birth
Peteca	Piaçava	F	11/2014
Duca	Dita	F	10/2014
Olívia	Doree	F	01/2015
Cacau	Chuchu	M	03/2015
Dançarina	Dita	F	02/2016
Oliveira	Doree	M	11/2016
Michele	Pamonha	F	12/2016
Dourado	Dita	M	03/2016
Pimenta	Piaçava	F	05/2017
Caititu	Chuchu	M	06/2017

### 2.3 Video Analysis

We screened the available footage of the focal subjects to identify all recordings containing events of infant food solicitation, defined here as any instance in which the infant attempted to grab food from the mother's mouth or hands when the mother was feeding (video footage showing this behavior is available in Supporting Material S1). For each video recording containing solicitation events, behavioral coding happened from the beginning to the end of the video. Across 194 h of footage, we identified 48 episodes in which one or more food solicitations occurred (dataset with monthly observation time, monthly number of solicitations and food sharing available in Supporting Material—Table S1; detailed dataset available in Supporting Material—in Table S2). Twenty‐five per cent of the videos were independently analyzed by three observers, and interobserver reliability was substantial (index of agreement Light's *kappa* = 0.70 between 3 observers). Interobserver agreement test incorporated all evaluated criteria based on: (1) the video timestamp that the solicitation occurred, (2) the food item and (3) success or failure in obtaining food.

Infant attempts to obtain food were scored as a ‘success’ whenever the infant succeeded in obtaining food and as a ‘failure’ whenever the infant did not get any food. We also registered the food items that were being consumed in every episode. To test our hypothesis, we classified the food items regarding their quality (high/low) and the level of difficulty to process (difficult/easy) based on the available literature (Table 2). We considered nuts and seeds as high‐quality items because they are a source of fats and carbohydrates, and have been proven to stabilize dietary macronutrient balance (Izar et al. 2022). Invertebrates and small vertebrates were classified as high‐quality, because of their high protein content (Izar et al. 2022). Even though fruits are usually considered high‐quality items (Brown et al. 2004), we classified them as low‐quality because they were high in carbohydrates but low in lipids and proteins. Other plant parts, such as branches, leaves and roots, were considered low‐quality. We classified nuts as difficult‐to‐process items because their consumption involves the use of tools (Fragaszy et al. 2004a; Fragaszy et al. 2017). Similarly, animals were categorized as difficult items because foraging for them demands dexterity and active pursuit of prey (Silk 1978). The remaining items were considered easy to process.

**Table 2:** Food items consumed at fazenda boa vista, their nutritional quality (high/low) and difficulty of processing (hard/easy), Based on Izar et al. (2022), Brown et al. (2004), and Silk (1978).

Food item	Nutritional quality	Difficulty of processing
Nuts	high	hard
Fruits	low	easy
Seeds	high	easy
Other plant parts	low	easy
Animals	high	hard

### 2.4 Statistical Analysis

Statistical analyses were performed using the software environment R v4.5.0. We fitted binomial Generalized Linear Mixed Models (GLMM) to investigate which factors influenced the likelihood of successful food transfers after infant solicitation. Infant transfer (success vs. failure) for each food item was used as our response variable in all of the models. We initially ran models with different combinations of variables. Fixed effects were evaluated through stepwise model selection based on the Akaike Information Criterion (AIC). Predictors with high multicollinearity (Variance Inflation Factor > 2) were excluded; collinearity was calculated with the ‘car’ package in R (Fox et al. 2012). Statistical analyses script is detailed on Supporting Material S2.

### 2.5 Ethical Note

The monkeys of this study were filmed without interference in their natural habitat in Brazil, except for the presence of human researchers. This study complied with protocols approved by the Animal Research Ethics Committee of the Institute of Psychology of the University of São Paulo (CEUA N 6870180216) and Brazilian legal requirements (ICMBio permit # 47501‐9).

## 3 Results

**Figure 1:**
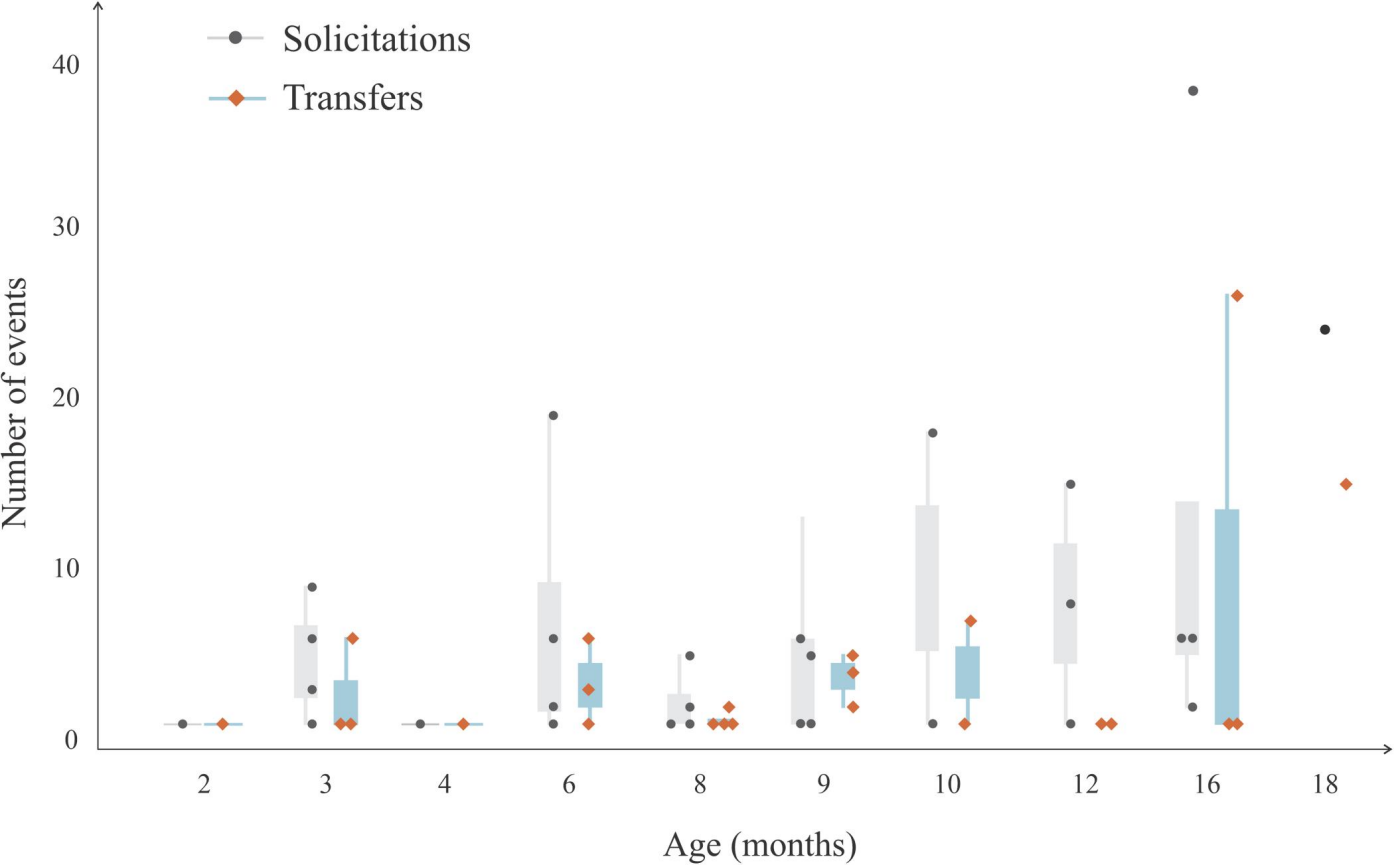
Distribution of food solicitation and transfer events based on observations of 10 infants from a wild population of *Sapajus libidinosus* at Fazenda Boa Vista (Brazil) during their first 18 months of life. Infants are highlighted as circles and diamonds.

Over 194 h of footage, we recorded 203 food solicitation events across 48 feeding episodes. The first solicitation and the first successful transfer both occurred in month 2 (only Oliveira) (Figure 1). Mothers shared food almost exclusively following infant solicitation. Only once did active sharing occur, when mother Doree gave a partially eaten palm nut to her 9‐month‐old infant Olivia, after multiple attempts from the infant to take the nut from the mother (Video on Supporting material S1). Infants successfully obtained food in 60% of bouts (29 successes, 19 failures). Of these, 48% (*n* = 14) involved no maternal resistance, while 52% (*n* = 15) required repeated solicitation. Out of the 203 solicitation events, 170 were from female infants with a success rate of 48.82% (83 transfers out 170), and 33 solicitations were from males with a success rate of 18.18% (6 transfers out of 33).

**Figure 2:**
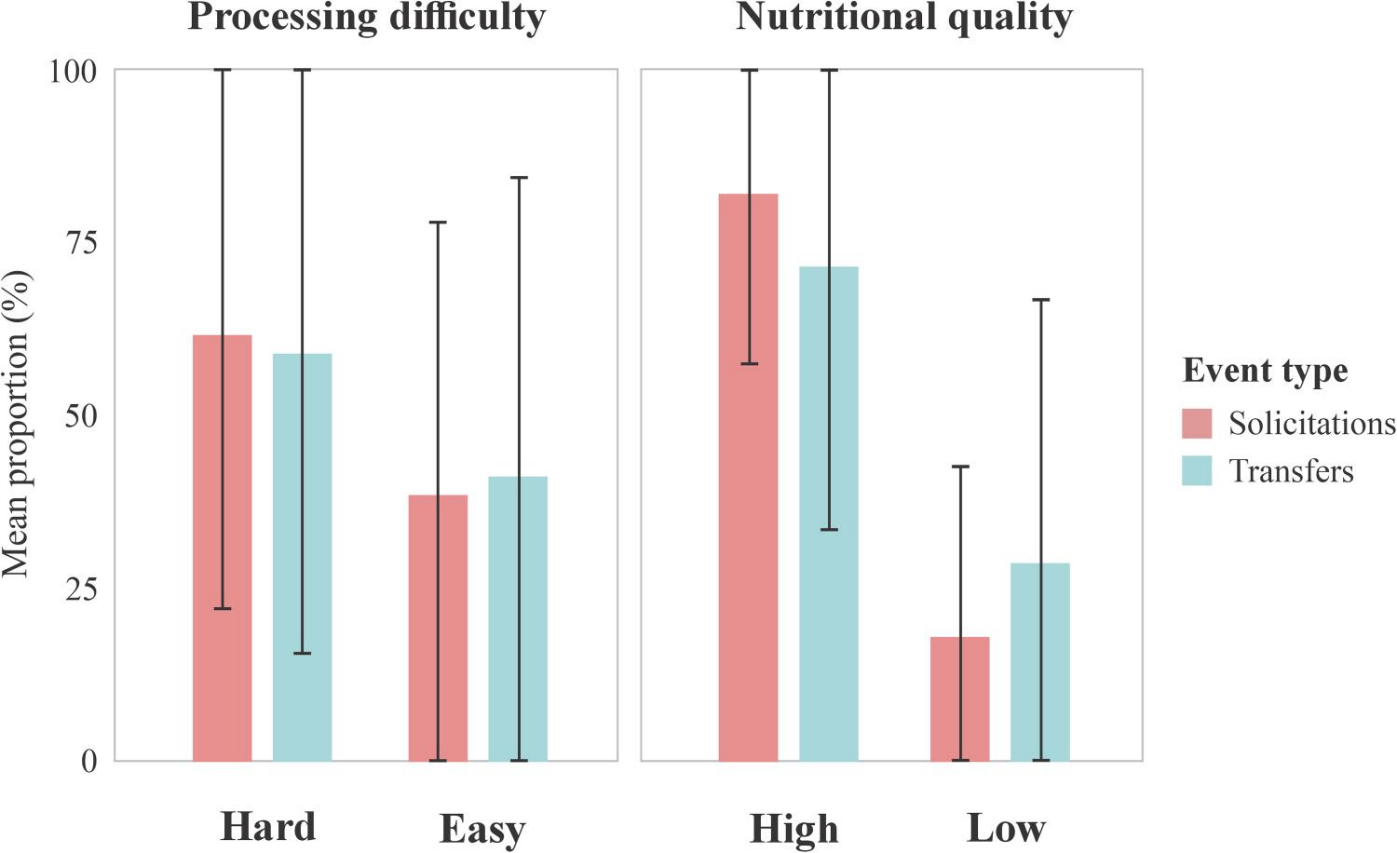
Mean proportion of food solicitation and transfer from mother to infant in relation to food nutritional quality and processing difficulty, based on observations of 10 infants from a wild population of *Sapajus libidinosus* at Fazenda Boa Vista, Brazil. Bars represent the average percentage of solicitation and successful transfer events across all observed infants. Error bars indicate standard deviation.

Among the 203 food solicitation events, infants predominantly targeted nuts (60.59%; *n* = 123) and lizards (11.33%; *n* = 23), both high‐quality and hard‐to‐process items. This population is known to prey on other vertebrates, such as birds and snakes (Izar et al. 2022; Verderane et al. 2013; Falótico et al. 2018). However, in our sample, the only vertebrate prey observed being consumed were lizards, recorded on two separate occasions, once by Dita and once by Doree. Fruits and other plant parts (low‐quality and easy‐to‐process items) were solicited in 26% of events (*n* = 54), while seeds (high‐quality and easy‐to‐process items) were rarely solicited (1%, *n* = 3). Overall, 89 of the 203 solicitations resulted in successful food transfers (43.84% of success rate); most of these were high‐quality and hard‐to‐process items (Figure 2).

A binomial GLMM including food nutritional quality or processing difficulty as a fixed effect, with infant identity as a random intercept, did not significantly predict transfer success (Model 1, Table 3). Because high‐quality foods were usually hard to process and low‐quality foods were usually easy, the two variables were strongly correlated (VIF > 10.0); we therefore included only one of them in the model. A second model that added food item, infant sex, mother identity, and infant age as fixed effects significantly improved model fit (χ^2^ = 35.31, df = 14, *p* < 0.001; all VIFs < 1.90). The final reduced model (all VIFs < 1.26), selected via a stepwise procedure, showed that transfer success was negatively associated with solicitations involving lizards (a vertebrate prey item), and positively associated with being the offspring of mother Doree and with increasing infant age (Model 2, Table 3). The model's predictive performance was moderate (ROC AUC = 0.74).

**Table 3:** GLMM results for predictors of transfer success.

Models	Fixed effects	*β*	*p*
Model 1	Nutritional quality/processing difficulty	0.26	0.47
Model 2	Lizard solicitation	–3.97	< 0.001
	Offspring of Doree	1.27	< 0.05
	Infant age	0.08	< 0.01
	Infant sex (male)	−0.74	0.32

## 4 Discussion

Our data provides support for the nutritional hypothesis (Brown et al. 2004): food sharing increases during the weaning period (from the 8th to the 16th month), when infants become less dependent on mother's milk and need nutritional support from other food sources. Infants also solicited more high‐quality and hard‐to‐process food items, indicating some support for both the nutritional and the informational hypothesis, but chances of sharing were not significantly higher for either food category. Food sharing always occurred in response to infant begging. Our data indicate that food sharing in capuchins is primarily influenced by the infant's behavioral repertoire and its nutritional and informational needs, rather than the mother's active parental investment.

As capuchin infants age, their rate of food solicitation increases. With physical development progressing (Fragaszy et al. 2016; Araujo et al. 2024), mothers begin to restrict access to nursing. Although nursing can continue until the second year of life, infants in this population are typically fully weaned by 18 months (Omena 2022). Notably, maternal rejections of nursing and transportation solicitations increase significantly by the sixth month (Cera et al. 2026), which may lead to increased food solicitation. In our study, we observed a significant rise in food‐sharing frequency in the sixth month compared to the fourth month of age. Solicitation rates also increased significantly from the eighth to the ninth month, a period during which infants may present a temporary behavioral regression to earlier developmental patterns (Verderane and Izar 2019). Begging behavior at this stage of development may indicate that individuals were hungrier due to a decrease in maternal milk intake and, therefore, attempted to obtain more solid food from their mothers. Also, during the feeding episodes, infants often touched or licked their mother's food; even if the food was not shared, this behavior likely contributes to the infant's learning about food sources through taste and tactile stimulation. As Ingold (2001) notes, skills are acquired through dynamic developmental processes. Similarly, capuchin monkeys are known to achieve proficiency at cracking nuts only after years of learning through their active interaction with objects and their environment (Resende et al. 2021). Therefore, manipulation of nuts processed by the mother may influence both learning processes and interest, ultimately facilitating the acquisition of tool‐use skills for nut cracking.

The pattern of food solicitation observed in this study aligns with findings in other primate species. For example, Javan gibbons (Yi et al. 2020) and orangutans (Jaeggi et al. 2008) also tend to solicit hard‐to‐process food items, similar to the capuchins at FBV. Our data reveals behavioral similarities between a Platyrrhine species and some Catarrhine primates, suggesting behavioral convergence in response to comparable environmental pressures. However, notable differences also emerge. Unlike capuchin monkeys, both gibbons and orangutans show a decline in food solicitation during the weaning period, and Javan gibbons do not exhibit a preference for high‐quality foods, instead favoring low‐quality or novel items, which supports the informational hypothesis. In contrast, capuchin infants frequently solicit nutritionally rich foods such as palm nuts and animals, which are high in lipids and proteins (Izar et al. 2022), indicating a nutritional motivation during weaning when milk intake in *Sapajus* decreases (Verderane and Izar 2019). Immature capuchins (*Sapajus* spp.) in captivity also have a preference for nuts, as observed by Fragaszy et al. (1997), indicating that this pattern of food solicitation by young monkeys remains similar across different robust capuchin species even when living in such different environments (captivity vs wild).

Interestingly, capuchin mothers were less likely to share lizards, possibly due to their rarity when compared to other food items (Izar et al. 2022). This could also indicate some level of protection of the infant against dangerous food types like lizards and other vertebrates that can harm a small infant if it is still alive or if it is poisonous. Adult meerkats are known for removing the stingers when provisioning live scorpions to young, inexperienced pups (Thornton and McAuliffe 2006). This hypothesis could be benefited from further research with a larger dataset on food solicitations of animal items and their toxicity to capuchin infants.

The unique episode of active food sharing, when the mother placed the food at the infant's feet, was the only instance of apparent proactive food sharing found in our data. This mother was Doree, the individual who was more likely to share food when compared to the other females of the study. Indeed, this behavior is rarely seen in the wild; passive transfers occur in captive capuchins (Fragaszy et al. 2004b), and we did not find any similar behavior on the 194 h of footage for the population we studied. In another species of capuchin monkey, *Sapajus nigritus*, only one instance of active food sharing was observed between a mother and her infant in a wild population studied for over two decades: the capuchin mother gave her partially eaten fruit to the infant carried on her back (P. Izar, pers. obs.). Among primates, proactive food sharing is most commonly observed in callitrichids. In these species, helpers of the breeding pair share high‐quality food, such as insects and other small prey, and even use specific vocalizations to attract immatures to the food source (Guerreiro Martins et al. 2019). Humans also actively share food, with the whole family typically contributing to feeding young children (Gurven 2004; Kaplan and Gurven 2005). Other primates are more likely to passively allow infants to take the food rather than provisioning them (Jaeggi et al. 2008; Nishida and Turner 1996; Yi et al. 2020). This highlights the contrast in food‐sharing strategies between species with cooperative offspring care, such as callitrichids and humans, and those with non‐cooperative care, where mothers are typically the primary caregivers (Burkart et al. 2017; Solomon and French 1997).

Although we found evidence that food sharing in bearded capuchins is primarily driven by infant solicitation, the role of the mother in these interactions is also noteworthy. In our descriptive analyses, we found that male infants were less likely than females to successfully obtain food following solicitation attempts. While the sex difference was not statistically significant in the models tested, a larger dataset could provide more precise estimates. We believe that this result can serve as a basis for generating new hypotheses, highlighting the need for further research on the topic. In primates, maternal investment often differs by offspring sex, typically favoring the phylopatric sex, the one that remains in the natal group after sexual maturity (Maestripieri 2018). In capuchin monkeys, females are the phylopatric sex (Izar et al. 2012), and immature females usually spend more time in social activities and initiate social behaviors earlier in development compared to males (Omena and Izar 2023). These developmental and social differences may underlie the observed sex bias in maternal food sharing: female infants, by spending more time in proximity to their mothers during early life, may have more opportunities to solicit food and thus achieve greater success in their attempts. We did not observe any instances of infants attempting to obtain food from individuals other than their mothers, although this behavior has been observed in this population by another researcher (B. Felicio, pers. obs., 2026). Fragaszy et al. (1997) noted that capuchins in captivity do attempt to take food from other peers and are successful in about 35% of their attempts. The wild population of this study is tolerant towards infants and young monkeys (Verderane et al. 2013), but more studies are necessary to assess the rates of solicitation and success of food transfers between infants and other group individuals that are not the mother.

Our findings offer novel insights into the role of infant behavior in the food‐sharing process of bearded capuchin monkeys (*S. libidinosus*). This study emphasizes the central role of infant behavior in eliciting food transfers. The increased frequency of food sharing during the weaning period, particularly in response to solicitations for high‐quality and hard‐to‐process items, suggests that the infant's nutritional needs are key drivers of this behavior. Observations of maternal selectivity, sex‐based differences in food sharing success, and the apparent avoidance of sharing vertebrate prey further suggest that maternal responses are context sensitive. Nevertheless, the limited number of observed sharing events constrains our ability to draw definitive conclusions. To better understand the complex dynamics underlying food sharing in capuchin monkeys, especially the interplay between nutritional, informational, and protective factors, future studies based on larger datasets are needed.

## Author Contributions

**Julia Omena:** conceptualization, investigation, writing – original draft, methodology, validation, visualization, formal analysis. **Mábia B. Cera:** conceptualization, investigation, writing – original draft, methodology, validation, visualization, formal analysis. **Guilbert Araujo:** investigation, writing – original draft, validation, visualization, formal analysis, software, methodology. **Helena F. Lima:** conceptualization, investigation, methodology. **Patrícia Izar:** conceptualization, investigation, funding acquisition, writing – review and editing, methodology, project administration, data curation, supervision, resources.

## Supporting information


**Video S1:** Footage of dyad Doree/Olivia showing the behaviors of food solicitation and food sharing.

Supporting File 1

Supporting File 2

Supporting File 3

## Data Availability

The data that supports the findings of this study are available in the Supporting Information of this article.
