# Supplementary material for: Can I Have a Bite? The Influence of Infant Begging on Food Sharing in Capuchin Monkeys (Sapajus Libidinosus)
Source: Am J Primatol. 2026 Jun 5;88(6):e70174. doi: 10.1002/ajp.70174 (PMC13238302; doi:10.1002/ajp.70174)
Supplement: Supplementary file 2 — Supporting File 1 [file AJP-88-e70174-s003.html]

Can I have a bite? The influence of infant begging on food sharing in capuchin monkeys (Sapajus libidinosus)


# Can I have a bite? The influence of infant begging on food sharing in capuchin monkeys (Sapajus libidinosus)

# 1 Statistical analyses

This report presents the statistical modeling conducted for the study “Can I have a bite? The influence of infant begging on food sharing in capuchin monkeys (*Sapajus libidinosus*)”. All analyses were performed in R (version 4.5.0).

# 2 Dataset description

The dataset includes 203 observations of 10 *Sapajus libidinosus* infants, followed from birth to 18 months using focal-animal sampling (Altmann, 1974). Videos were recorded weekly from dawn to dusk and are archived at **Laboratory of Ethology, Development, and Social Interaction (LEDIS)** at the Institute of Psychology, University of São Paulo. We selected timepoints across infancy (2nd to 18th month, every other month) and focused on feeding bouts involving food solicitation. Each event was coded for transfer success (yes/no), food quality (high/low), and processing difficulty (hard/easy), based on established literature. The dataset includes variables on infant identity, mother, age, sex, food item, and the binary outcomes described. All categorical variables were converted to factors for analysis.

```
summary(dados_2[, c("month","sex", "infant", "mother", "transfer", "quality", "processing", "item")])
```

```
     month       sex        infant             mother          transfer quality
 Min.   : 2.00   F:170   Length:203         Length:203         0:114    0: 54  
 1st Qu.: 8.00   M: 33   Class :character   Class :character   1: 89    1:149  
 Median :10.00           Mode  :character   Mode  :character                   
 Mean   :11.23                                                                 
 3rd Qu.:16.00                                                                 
 Max.   :18.00                                                                 
 processing     item          
 0: 57      Length:203        
 1:146      Class :character  
            Mode  :character
```

# 3 Adjusting Models

To investigate which factors influence the likelihood of food transfer, we fitted a series of binomial Generalized Linear Models (GLMs) and Generalized Linear Mixed Models (GLMMs) – including infant as a random effect.

## 3.1 Model 1

We first tested a full model including both quality and processing as fixed effects. Variance Inflation Factors (VIF) were examined to assess multicollinearity. Since processing contributed little to the model, we fitted a reduced model (modelo\_1.2) including only quality. However, this reduced model did not show a significant improvement over the null model.

### 3.1.1 Null model

```
null_model <- glmer(transfer ~ 1 + (1 | infant),
                    data = dados_2,
                    family = binomial)
summ(null_model)
```

|  |  |
| --- | --- |
| Observations | 203 |
| Dependent variable | transfer |
| Type | Mixed effects generalized linear model |
| Family | binomial |
| Link | logit |

|  |  |
| --- | --- |
| AIC | 277.97 |
| BIC | 284.59 |
| Pseudo-R² (fixed effects) | 0.00 |
| Pseudo-R² (total) | 0.15 |

| Fixed Effects | | | | |
| --- | --- | --- | --- | --- |
|  | Est. | S.E. | z val. | p |
| (Intercept) | -0.64 | 0.38 | -1.69 | 0.09 |

| Random Effects | | |
| --- | --- | --- |
| Group | Parameter | Std. Dev. |
| infant | (Intercept) | 0.76 |

| Grouping Variables | | |
| --- | --- | --- |
| Group | # groups | ICC |
| infant | 10 | 0.15 |

### 3.1.2 Model including ‘quality’ and ‘processing’ as fixed effects

```
modelo_1 <- glmer(transfer ~ quality + processing + (1 | infant), 
                  data = dados_2, 
                  family = binomial)

summ(modelo_1)
```

|  |  |
| --- | --- |
| Observations | 203 |
| Dependent variable | transfer |
| Type | Mixed effects generalized linear model |
| Family | binomial |
| Link | logit |

|  |  |
| --- | --- |
| AIC | 281.45 |
| BIC | 294.70 |
| Pseudo-R² (fixed effects) | 0.00 |
| Pseudo-R² (total) | 0.16 |

| Fixed Effects | | | | |
| --- | --- | --- | --- | --- |
|  | Est. | S.E. | z val. | p |
| (Intercept) | -0.85 | 0.49 | -1.72 | 0.09 |
| quality1 | 0.20 | 1.43 | 0.14 | 0.89 |
| processing1 | 0.06 | 1.40 | 0.04 | 0.97 |

| Random Effects | | |
| --- | --- | --- |
| Group | Parameter | Std. Dev. |
| infant | (Intercept) | 0.78 |

| Grouping Variables | | |
| --- | --- | --- |
| Group | # groups | ICC |
| infant | 10 | 0.16 |

### 3.1.3 Variance Inflation Factor (VIF)

```
vif(modelo_1)
```

```
   quality processing 
  15.24273   15.24273
```

### 3.1.4 Reduced model

```
modelo_1.2 <- glmer(transfer ~ quality + (1 | infant), 
                    data = dados_2, 
                    family = binomial)

summ(modelo_1.2)
```

|  |  |
| --- | --- |
| Observations | 203 |
| Dependent variable | transfer |
| Type | Mixed effects generalized linear model |
| Family | binomial |
| Link | logit |

|  |  |
| --- | --- |
| AIC | 279.45 |
| BIC | 289.39 |
| Pseudo-R² (fixed effects) | 0.00 |
| Pseudo-R² (total) | 0.16 |

| Fixed Effects | | | | |
| --- | --- | --- | --- | --- |
|  | Est. | S.E. | z val. | p |
| (Intercept) | -0.85 | 0.49 | -1.74 | 0.08 |
| quality1 | 0.26 | 0.37 | 0.72 | 0.47 |

| Random Effects | | |
| --- | --- | --- |
| Group | Parameter | Std. Dev. |
| infant | (Intercept) | 0.79 |

| Grouping Variables | | |
| --- | --- | --- |
| Group | # groups | ICC |
| infant | 10 | 0.16 |

### 3.1.5 Comparison between reduced and null models (Chi-Squared Test)

```
anova(null_model, modelo_1.2, test = "Chisq")
```

```
Data: dados_2
Models:
null_model: transfer ~ 1 + (1 | infant)
modelo_1.2: transfer ~ quality + (1 | infant)
           npar    AIC    BIC  logLik -2*log(L)  Chisq Df Pr(>Chisq)
null_model    2 277.97 284.59 -136.98    273.97                     
modelo_1.2    3 279.45 289.39 -136.72    273.45 0.5175  1     0.4719
```

## 3.2 Model 2

In this section, we fitted a logistic regression model to investigate the effects of various predictors on the likelihood of food transfer. We started by including all relevant variables in a generalized linear model with a binomial family. Multicollinearity among predictors was assessed using Variance Inflation Factors (VIF), and stepwise model selection based on AIC was applied to identify the most parsimonious model. Model fit was further evaluated by checking overdispersion. To assess predictive performance, we constructed a confusion matrix and plotted the Receiver Operating Characteristic (ROC) curve, calculating the Area Under the Curve (AUC). Although a mixed-effects model including infant identity as a random effect was initially considered, the difference in AIC between this model and the fixed-effects model was minimal (≤ 2), indicating comparable model fit.

### 3.2.1 Null model

```
null_model <- glmer(transfer ~ 1 + (1 | infant),
                    data = dados_2,
                    family = binomial)
summ(null_model)
```

|  |  |
| --- | --- |
| Observations | 203 |
| Dependent variable | transfer |
| Type | Mixed effects generalized linear model |
| Family | binomial |
| Link | logit |

|  |  |
| --- | --- |
| AIC | 277.97 |
| BIC | 284.59 |
| Pseudo-R² (fixed effects) | 0.00 |
| Pseudo-R² (total) | 0.15 |

| Fixed Effects | | | | |
| --- | --- | --- | --- | --- |
|  | Est. | S.E. | z val. | p |
| (Intercept) | -0.64 | 0.38 | -1.69 | 0.09 |

| Random Effects | | |
| --- | --- | --- |
| Group | Parameter | Std. Dev. |
| infant | (Intercept) | 0.76 |

| Grouping Variables | | |
| --- | --- | --- |
| Group | # groups | ICC |
| infant | 10 | 0.15 |

### 3.2.2 Fitting the Binomial Model

```
model_2_glmm <- glmer(transfer ~ sex + item_fruit + item_insect + item_leaf + item_lizard + item_mango + item_nut + item_root + item_seed + item_twig + item_vertebrate + mother_Dita + mother_Doree + mother_Pamonha + mother_Piaçava + month + (1 | infant), 
                        data = dados_2_dummies, 
                        family = binomial)

model_2_glm <- glm(transfer ~ sex + item_fruit + item_insect + item_leaf + item_lizard + item_mango + item_nut + item_root + item_seed + item_twig + item_vertebrate + mother_Dita + mother_Doree + mother_Pamonha + mother_Piaçava + month, 
                        data = dados_2_dummies, 
                        family = binomial)

AIC(model_2_glm, model_2_glmm)
```

```
             df      AIC
model_2_glm  17 271.5845
model_2_glmm 18 273.5845
```

```
vif(model_2_glm)
```

```
            sex      item_fruit     item_insect       item_leaf     item_lizard 
       7.456989        1.448357        1.616753        1.346283        3.103780 
     item_mango        item_nut       item_root       item_seed       item_twig 
       2.340769        4.607813        1.706149        1.285464        1.323554 
item_vertebrate     mother_Dita    mother_Doree  mother_Pamonha  mother_Piaçava 
       1.993055        3.963369       12.493852       24.496650       29.492830 
          month 
       3.127292
```

```
step <- step(object = model_2_glm,
             k = qchisq(p = 0.05, df = 1, lower.tail = FALSE))
```

```
Start:  AIC=302.89
transfer ~ sex + item_fruit + item_insect + item_leaf + item_lizard + 
    item_mango + item_nut + item_root + item_seed + item_twig + 
    item_vertebrate + mother_Dita + mother_Doree + mother_Pamonha + 
    mother_Piaçava + month

                  Df Deviance    AIC
- item_seed        1   237.62 299.08
- item_vertebrate  1   237.68 299.14
- mother_Dita      1   237.89 299.36
- item_mango       1   238.00 299.46
- sex              1   238.03 299.50
- item_insect      1   238.10 299.57
- item_twig        1   239.13 300.60
- mother_Piaçava   1   239.15 300.61
- item_leaf        1   239.59 301.06
- item_nut         1   239.88 301.35
- item_fruit       1   240.49 301.95
- mother_Pamonha   1   240.69 302.15
- item_root        1   240.71 302.17
<none>                 237.58 302.89
- mother_Doree     1   242.90 304.36
- item_lizard      1   243.05 304.52
- month            1   244.48 305.94

Step:  AIC=299.08
transfer ~ sex + item_fruit + item_insect + item_leaf + item_lizard + 
    item_mango + item_nut + item_root + item_twig + item_vertebrate + 
    mother_Dita + mother_Doree + mother_Pamonha + mother_Piaçava + 
    month

                  Df Deviance    AIC
- item_vertebrate  1   237.77 295.39
- item_mango       1   238.00 295.62
- mother_Dita      1   238.01 295.63
- item_insect      1   238.11 295.74
- sex              1   238.20 295.82
- item_twig        1   239.14 296.76
- mother_Piaçava   1   239.36 296.98
- item_leaf        1   239.60 297.23
- item_nut         1   239.93 297.55
- item_fruit       1   240.49 298.12
- item_root        1   240.72 298.34
- mother_Pamonha   1   240.90 298.52
<none>                 237.62 299.08
- mother_Doree     1   243.48 301.10
- month            1   244.69 302.31
- item_lizard      1   244.87 302.49

Step:  AIC=295.39
transfer ~ sex + item_fruit + item_insect + item_leaf + item_lizard + 
    item_mango + item_nut + item_root + item_twig + mother_Dita + 
    mother_Doree + mother_Pamonha + mother_Piaçava + month

                 Df Deviance    AIC
- mother_Dita     1   238.04 291.82
- sex             1   238.20 291.98
- item_mango      1   238.24 292.02
- item_insect     1   238.30 292.08
- item_twig       1   239.33 293.11
- mother_Piaçava  1   239.41 293.19
- item_leaf       1   239.78 293.56
- item_nut        1   240.26 294.05
- item_fruit      1   240.67 294.45
- item_root       1   240.94 294.72
- mother_Pamonha  1   241.02 294.80
<none>                237.77 295.39
- mother_Doree    1   244.35 298.13
- month           1   244.74 298.52
- item_lizard     1   246.69 300.47

Step:  AIC=291.82
transfer ~ sex + item_fruit + item_insect + item_leaf + item_lizard + 
    item_mango + item_nut + item_root + item_twig + mother_Doree + 
    mother_Pamonha + mother_Piaçava + month

                 Df Deviance    AIC
- sex             1   238.22 288.16
- item_mango      1   238.44 288.37
- item_insect     1   238.45 288.39
- item_twig       1   239.45 289.39
- item_leaf       1   239.86 289.80
- mother_Piaçava  1   240.08 290.02
- item_nut        1   240.28 290.22
- item_fruit      1   240.79 290.73
- item_root       1   241.02 290.96
<none>                238.04 291.82
- mother_Pamonha  1   243.99 293.92
- month           1   244.78 294.71
- item_lizard     1   247.71 297.65
- mother_Doree    1   250.94 300.88

Step:  AIC=288.16
transfer ~ item_fruit + item_insect + item_leaf + item_lizard + 
    item_mango + item_nut + item_root + item_twig + mother_Doree + 
    mother_Pamonha + mother_Piaçava + month

                 Df Deviance    AIC
- item_mango      1   238.53 284.63
- item_insect     1   238.56 284.66
- item_twig       1   239.54 285.63
- item_leaf       1   239.98 286.08
- item_nut        1   240.28 286.38
- mother_Piaçava  1   240.57 286.67
- item_fruit      1   240.86 286.96
- item_root       1   241.03 287.13
<none>                238.22 288.16
- mother_Pamonha  1   245.49 291.59
- month           1   245.77 291.87
- item_lizard     1   248.47 294.56
- mother_Doree    1   251.21 297.31

Step:  AIC=284.63
transfer ~ item_fruit + item_insect + item_leaf + item_lizard + 
    item_nut + item_root + item_twig + mother_Doree + mother_Pamonha + 
    mother_Piaçava + month

                 Df Deviance    AIC
- item_insect     1   238.70 280.95
- item_twig       1   239.61 281.87
- item_leaf       1   240.08 282.34
- item_nut        1   240.60 282.86
- item_fruit      1   240.96 283.21
- item_root       1   241.03 283.29
- mother_Piaçava  1   241.11 283.37
<none>                238.53 284.63
- mother_Pamonha  1   245.50 287.76
- month           1   246.79 289.05
- mother_Doree    1   251.66 293.92
- item_lizard     1   252.63 294.89

Step:  AIC=280.95
transfer ~ item_fruit + item_leaf + item_lizard + item_nut + 
    item_root + item_twig + mother_Doree + mother_Pamonha + mother_Piaçava + 
    month

                 Df Deviance    AIC
- item_twig       1   239.68 278.09
- item_leaf       1   240.13 278.55
- item_nut        1   240.60 279.02
- item_fruit      1   241.01 279.42
- item_root       1   241.04 279.45
- mother_Piaçava  1   241.42 279.83
<none>                238.70 280.95
- mother_Pamonha  1   245.51 283.92
- month           1   246.80 285.21
- mother_Doree    1   251.68 290.09
- item_lizard     1   253.12 291.54

Step:  AIC=278.09
transfer ~ item_fruit + item_leaf + item_lizard + item_nut + 
    item_root + mother_Doree + mother_Pamonha + mother_Piaçava + 
    month

                 Df Deviance    AIC
- item_leaf       1   240.91 275.49
- item_nut        1   241.14 275.71
- item_root       1   241.68 276.26
- item_fruit      1   241.70 276.27
- mother_Piaçava  1   242.56 277.13
<none>                239.68 278.09
- mother_Pamonha  1   246.32 280.89
- month           1   247.16 281.74
- mother_Doree    1   252.32 286.90
- item_lizard     1   254.43 289.01

Step:  AIC=275.49
transfer ~ item_fruit + item_lizard + item_nut + item_root + 
    mother_Doree + mother_Pamonha + mother_Piaçava + month

                 Df Deviance    AIC
- item_nut        1   242.13 272.86
- item_root       1   242.54 273.28
- item_fruit      1   242.69 273.42
- mother_Piaçava  1   244.11 274.84
<none>                240.91 275.49
- mother_Pamonha  1   247.02 277.75
- month           1   247.31 278.04
- mother_Doree    1   253.00 283.74
- item_lizard     1   255.37 286.10

Step:  AIC=272.86
transfer ~ item_fruit + item_lizard + item_root + mother_Doree + 
    mother_Pamonha + mother_Piaçava + month

                 Df Deviance    AIC
- item_root       1   243.23 270.12
- item_fruit      1   243.59 270.48
- mother_Piaçava  1   245.06 271.95
<none>                242.13 272.86
- mother_Pamonha  1   247.62 274.51
- month           1   251.11 278.00
- mother_Doree    1   254.44 281.33
- item_lizard     1   264.62 291.51

Step:  AIC=270.12
transfer ~ item_fruit + item_lizard + mother_Doree + mother_Pamonha + 
    mother_Piaçava + month

                 Df Deviance    AIC
- item_fruit      1   244.52 267.57
- mother_Piaçava  1   246.18 269.23
<none>                243.23 270.12
- mother_Pamonha  1   248.09 271.13
- month           1   251.17 274.22
- mother_Doree    1   254.73 277.78
- item_lizard     1   265.10 288.15

Step:  AIC=267.57
transfer ~ item_lizard + mother_Doree + mother_Pamonha + mother_Piaçava + 
    month

                 Df Deviance    AIC
- mother_Piaçava  1   247.34 266.55
<none>                244.52 267.57
- mother_Pamonha  1   249.95 269.16
- month           1   251.48 270.69
- mother_Doree    1   255.31 274.52
- item_lizard     1   265.88 285.09

Step:  AIC=266.55
transfer ~ item_lizard + mother_Doree + mother_Pamonha + month

                 Df Deviance    AIC
- mother_Pamonha  1   249.95 265.32
<none>                247.34 266.55
- mother_Doree    1   255.59 270.96
- month           1   257.00 272.37
- item_lizard     1   275.02 290.39

Step:  AIC=265.32
transfer ~ item_lizard + mother_Doree + month

               Df Deviance    AIC
<none>              249.95 265.32
- mother_Doree  1   255.93 267.45
- month         1   257.06 268.59
- item_lizard   1   275.94 287.46
```

```
summ(step, confint = T, digits = 3, ci.width = .95)
```

|  |  |
| --- | --- |
| Observations | 203 |
| Dependent variable | transfer |
| Type | Generalized linear model |
| Family | binomial |
| Link | logit |

|  |  |
| --- | --- |
| χ²(3) | 28.380 |
| p | 0.000 |
| Pseudo-R² (Cragg-Uhler) | 0.175 |
| Pseudo-R² (McFadden) | 0.102 |
| AIC | 257.951 |
| BIC | 271.204 |

|  | Est. | 2.5% | 97.5% | z val. | p |
| --- | --- | --- | --- | --- | --- |
| (Intercept) | -1.120 | -1.917 | -0.323 | -2.754 | 0.006 |
| item\_lizard | -3.965 | -6.169 | -1.761 | -3.526 | 0.000 |
| mother\_Doree | 1.279 | 0.213 | 2.346 | 2.351 | 0.019 |
| month | 0.084 | 0.021 | 0.147 | 2.617 | 0.009 |
|  |  |  |  |  |  |
| --- | --- | --- | --- | --- | --- |
| Standard errors: MLE |  |  |  |  |  |

### 3.2.3 Variance Inflation Factor (VIF)

```
vif(step)
```

```
 item_lizard mother_Doree        month 
    1.172447     1.252367     1.093223
```

```
dispersion <- sum(residuals(step, type = "deviance")^2) / step$df.residual
dispersion
```

```
[1] 1.256035
```

### 3.2.5 Residuals

```
#plot residuos
 par(mfrow=c(2,2))
 plot(step, pch=19, col="blue")
```

### 3.2.6 ROC curve

```
# Confusion Matrix
confusionMatrix(table(predict(model_2_glm, type = "response") >= 0.5, 
                      dados_2_dummies$transfer == "1")[2:1, 2:1])
```

```
Confusion Matrix and Statistics

       
        TRUE FALSE
  TRUE    62    34
  FALSE   27    80
                                          
               Accuracy : 0.6995          
                 95% CI : (0.6314, 0.7617)
    No Information Rate : 0.5616          
    P-Value [Acc > NIR] : 3.792e-05       
                                          
                  Kappa : 0.395           
                                          
 Mcnemar's Test P-Value : 0.4424          
                                          
            Sensitivity : 0.6966          
            Specificity : 0.7018          
         Pos Pred Value : 0.6458          
         Neg Pred Value : 0.7477          
             Prevalence : 0.4384          
         Detection Rate : 0.3054          
   Detection Prevalence : 0.4729          
      Balanced Accuracy : 0.6992          
                                          
       'Positive' Class : TRUE
```

```
# ROC Curve
ROC <- roc(response = dados_2_dummies$transfer, 
           predictor = model_2_glm$fitted.values)


ggplotly(
  ggroc(ROC, color = "darkorchid", linewidth = 1) +  
    annotate("segment", x = 1, xend = 0, y = 0, yend = 1, 
             color = "orange", linewidth = 0.2) +     
    labs(
      x = "1 - Specificity",
      y = "Sensitivity",
      title = paste("Area Under the Curve:", 
                    round(ROC$auc, 3), 
                    "|",
                    "Gini Coefficient:", 
                    round((ROC$auc[1] - 0.5) / 0.5, 3))
    ) +
    theme_bw()
)
```
